# Modeling key pathological features of frontotemporal dementia with *C9ORF72* repeat expansion in iPSC-derived human neurons

**DOI:** 10.1007/s00401-013-1149-y

**Published:** 2013-07-09

**Authors:** Sandra Almeida, Eduardo Gascon, Hélène Tran, Hsin Jung Chou, Tania F. Gendron, Steven DeGroot, Andrew R. Tapper, Chantal Sellier, Nicolas Charlet-Berguerand, Anna Karydas, William W. Seeley, Adam L. Boxer, Leonard Petrucelli, Bruce L. Miller, Fen-Biao Gao

**Affiliations:** 1 Department of Neurology, University of Massachusetts Medical School, Worcester, MA 01605 USA; 2 Department of Neuroscience, Mayo Clinic Florida, Jacksonville, FL 32224 USA; 3 Department of Psychiatry, Brudnick Neuropsychiatric Research Institute, University of Massachusetts Medical School, Worcester, MA 01604 USA; 4 Department of Neurobiology and Genetics, IGBMC, INSERM U964, CNRS UMR7104, University of Strasbourg, Illkirch, France; 5 Department of Neurology, Memory and Aging Center, University of California, San Francisco, CA 94143 USA

**Keywords:** ALS, Autophagy, C9ORF72, FTD, Hexanucleotide repeats, iPSCs, Neurodegeneration, Neurons, p62, RAN translation, RNA foci

## Abstract

The recently identified GGGGCC repeat expansion in the noncoding region of *C9ORF72* is the most common pathogenic mutation in patients with frontotemporal dementia (FTD) or amyotrophic lateral sclerosis (ALS). We generated a human neuronal model and investigated the pathological phenotypes of human neurons containing GGGGCC repeat expansions. Skin biopsies were obtained from two subjects who had >1,000 GGGGCC repeats in *C9ORF72* and their respective fibroblasts were used to generate multiple induced pluripotent stem cell (iPSC) lines. After extensive characterization, two iPSC lines from each subject were selected, differentiated into postmitotic neurons, and compared with control neurons to identify disease-relevant phenotypes. Expanded GGGGCC repeats exhibit instability during reprogramming and neuronal differentiation of iPSCs. RNA foci containing GGGGCC repeats were present in some iPSCs, iPSC-derived human neurons and primary fibroblasts. The percentage of cells with foci and the number of foci per cell appeared to be determined not simply by repeat length but also by other factors. These RNA foci do not seem to sequester several major RNA-binding proteins. Moreover, repeat-associated non-ATG (RAN) translation products were detected in human neurons with GGGGCC repeat expansions and these neurons showed significantly elevated p62 levels and increased sensitivity to cellular stress induced by autophagy inhibitors. Our findings demonstrate that key neuropathological features of FTD/ALS with GGGGCC repeat expansions can be recapitulated in iPSC-derived human neurons and also suggest that compromised autophagy function may represent a novel underlying pathogenic mechanism.

**Electronic supplementary material:**

The online version of this article (doi:10.1007/s00401-013-1149-y) contains supplementary material, which is available to authorized users.

## Introduction

Frontotemporal dementia (FTD), the second most common form of presenile dementia, is associated with focal atrophy of the frontal or temporal lobes [9]. FTD shares extensive clinical, pathological, and molecular overlap with amyotrophic lateral sclerosis (ALS), a neurodegenerative disease that has devastating effects on motor neurons in the spinal cord and on frontal neurons in the brain [37]. Strikingly, the RNA-binding proteins, TDP-43 and FUS, are major pathological proteins in both FTD and ALS [6, 26, 33, 34, 45]. Moreover, both FTD and ALS can be caused by mutations in the same gene, such as those encoding valosin-containing protein (VCP) [22, 46], charged multivesicular body protein 2B (CHMP2B) [11, 42], ubiquilin 2 (UBQLN2) [14], and chromosome 9 open reading frame 72 (C9ORF72) [12, 39].

The GGGGCC repeat expansion in the noncoding region of *C9ORF72* is the most common known pathogenic mutation in FTD and ALS [12, 30, 39]. *C9ORF72* encodes an uncharacterized protein, and it is not known how GGGGCC repeat expansions cause FTD/ALS. It has been proposed that *C9ORF72* haploinsufficiency, RNA toxicity, or both are potential pathogenic mechanisms in patients with *C9ORF72* repeat expansions [12, 19, 39]. Whether RNA foci are present in neurons of FTD and ALS patients with GGGGCC repeat expansions is controversial [12, 41]. Recently, repeat-associated non-ATG (RAN) translation has been detected in a subset of neurons in patient brains, raising the possibility that neurotoxicity of di-peptide repeats may be a third major pathogenic mechanism in these patients [7, 32].

So far, understanding of pathogenic mechanisms has been hampered by the lack of suitable cellular or animal model of GGGGCC repeat expansion. Traditional approaches to disease modeling have a number of potential intrinsic limitations, for instance, a disease gene is often overexpressed. Moreover, long repeat sequences are often unstable, posing a significant technical challenge for molecular cloning and disease modeling of *C9ORF72*-related FTD/ALS in animals. Induced pluripotent stem cell (iPSC) technology allows mechanistic studies of disease genes in their native genetic contexts [49]. Indeed, iPSC models of several neurodegenerative diseases have been established [3, 15, 16, 20, 21, 24, 35, 43]. In this study, we generated multiple iPSC lines from two members of the Vancouver, San Francisco, and Mayo family 20 (VSM-20 family) [12] with long GGGGCC repeats and investigated the neuropathological features and novel disease mechanisms in patient-derived iPSCs and human neurons.

## Materials and methods

### Human subjects

Skin biopsies were obtained from two VSM-20 family members, one with predominantly bvFTD, and the other a presymptomatic carrier. To protect their privacy, they are simply named as carrier 1 and carrier 2 without disclosing their personal information. Frozen tissues, consisting of 1 cm^3^ blocks of superior frontal gyrus, were obtained from two middle-aged males with bvFTD due to *C9ORF72* hexanucleotide repeat expansion. The study was approved by the Institutional Review Board and Ethics Committees at the University of California, San Francisco (UCSF) and written informed consent was obtained from all participants in this study.

### Isolation of primary human skin fibroblasts and generation of iPSCs

Skin biopsies were cut into small pieces and placed on culture dishes to allow fibroblasts to expand. The cells were maintained in Dulbecco’s modified Eagle’s medium supplemented with 10 % fetal bovine serum, 1X nonessential amino acids, and penicillin/streptomycin (100 U/ml).

FTD patient-specific iPSCs were generated as described [3, 44]. Briefly, fibroblasts (8 × 10^5^ per 100 mm dish) were transduced with equal volumes of supernatants from cultures of retroviruses expressing human *OCT3/4*, *SOX2*, *KLF4,* and *c*-*MYC*. The next day, the medium was removed and replaced with fresh viral supernatants. Seven days after the first infection, cells were collected and seeded (5 × 10^4^ cells per 100 mm dish) on SNL feeder cells treated with mitomycin C. One day later, the medium was replaced with iPSC medium containing 4 ng/ml basic fibroblast growth factor; thereafter, the medium was changed every other day. Five weeks after viral transduction, colonies were picked, transferred to 12-well plates coated with Matrigel (BD Biosciences), and cultured in mTeSR1 medium (StemCell Technologies). For expansion, cells were dissociated with 1:2 accutase/PBS solution for 1 min at room temperature, washed twice with PBS, and scraped with a cell lifter into mTeSR1 medium. Larger colonies were further broken up by pipetting and transferred to 6-well plates.

### Neuronal differentiation and immunocytochemistry

Neuronal differentiation of human iPSC lines and immunocytochemistry on postmitotic neurons were performed as described [3].

For neuronal differentiation, iPSC colonies were detached with accutase (Millipore) and grown as embryoid bodies (EBs) in suspension for 5–6 days in iPSC medium in the absence of basic fibroblast growth factor. EBs were allowed to attach and form rosettes. Ten-day-old rosettes were collected and grown in suspension as neurospheres. Neurospheres were dissociated after 3–4 weeks, and the cells were placed on glass coverslips (BD Biosciences) or plates coated with poly-d-lysine (0.1 mg/ml) and laminin (10 μg/ml). Neurons were used after 2–4 weeks in culture.

For in vitro differentiation, EBs were generated as described above, grown for 8 days in suspension, placed on Matrigel-coated glass coverslips, and allowed to further differentiate for 8 days in mTeSR1 medium. Cells migrating out of the attached EBs were stained and analyzed by fluorescence microscopy (Olympus IX-70 microscope) for markers of the three germ layers. Karyotype analysis was done at the Cytogenetic Laboratory, UMASS Memorial (Worcester, MA), using standard protocols for G-banding.

For immunocytochemistry, cells were fixed in 4 % paraformaldehyde (pH 7.4) for 10 min and permeabilized with 0.2 % Triton X-100. After blocking with 3 % bovine serum albumin for 30 min, cells were incubated with primary antibodies for 1 h at room temperature or overnight at 4C. The primary antibodies were goat anti-Nanog (R&D Systems; 1:100), mouse anti-SSEA4 (Abcam; 1:100), rabbit anti-desmin (Thermo Scientific; 1:100), mouse anti-βIII-tubulin (Promega; 1:500), and mouse anti-α-fetoprotein (R&D Systems; 1:200), mouse anti-MAP2 (Sigma; 1:500), rabbit anti-glial fibrillary acidic protein (Dako; 1:1,000), rabbit anti-VGLUT1 (Synaptic Systems; 1:500), rabbit anti-GABA (Sigma; 1:100), rabbit anti-tyrosine hydroxylase (Millipore; 1:500), mouse anti-hnRNP A2/B1 (Santa Cruz Biotechnologies; 1:700), goat anti-hnRNP H1 (Santa Cruz Biotechnologies; 1:3,000), rabbit anti-hnRNP H2 (Sigma; 1:300), mouse anti-hnRNP F (Santa Cruz Biotechnologies; 1:1,000), rabbit anti-TDP43 (Protein Tech Group; 1:100), rabbit anti-FUS (Protein Tech Group; 1:300), mouse anti-nucleophosmin (Abcam; 1:3,000). After three washes with PBS, the cells were incubated with AlexaFluor-conjugated secondary antibodies (Invitrogen; 1:300) for 1 h at room temperature and counterstained with 1 μg/ml Hoechst or DAPI. Immunostained cells were examined by fluorescence microscopy.

### Electrophysiology

Neurons (3–4 weeks in culture) plated on coverslips were mounted onto on an upright microscope (BX51WI, Olympus). Neurons were bathed in pH 7.4 oxygenated extracellular solution containing: 125 mM NaCl, 2.5 mM KCl, 1.2 mM NaH_2_PO_4_, 1.2 mM MgCl_2_, 2.4 mM CaCl_2_, 26 mM NaHCO_3_, 11 mM d-Glucose. Neurons were visually identified via infrared (IR) differential interference contrast video microscopy with an IR-CCD camera (Olympus). Depolarization-evoked action potentials and spontaneous EPSCs were recorded at room temperature using the whole-cell configuration of a Multiclamp 700B patch-clamp amplifier (Molecular Devices) in current- or voltage-clamp mode, respectively. The junction potential between the patch pipette and extracellular solution was nullified just prior to obtaining a seal on the neuronal membrane. Internal pipette solution contained: 121 mM KCl, 4 mM MgCl_2_, 11 mM EGTA, 1 mM CaCl_2_, 10 mM HEPES, 0.2 mM GTP, and 4 mM ATP. Signals were filtered at 2 kHz using the amplifier’s four-pole, low-pass Bessel filter, digitized at 10 kHz with an Axon Digidata 1,440 A interface and stored on a personal computer. Extracellular solution with or without CNQX (10 μM) or TTX (0.5 μM) was applied onto neurons by gravity superfusion.

### qRT-PCR and northern blot

Total RNA was isolated using RNeasy Kit (Qiagen) and 500 ng of RNA were reverse transcribed to cDNA using TaqMan Reverse Transcription Reagents kit (Applied Biosystems) following the manufacturer’s instructions. Quantitative PCR was performed using SYBR Green Master Mix (Applied Biosystems) and 10 μM of forward and reverse primers or TaqMan Gene Expression Master Mix and TaqMan primers (*C9ORF72* variant 1, Applied Biosystems). Ct values for each sample and gene were normalized to *GAPDH* gene. The 2exp (−ΔΔCt) method was used to determine the relative expression of each gene. Primers used in this study can be found in Table S1. Analysis of the transgenes silencing was performed as described [3].

For northern blot analysis, total RNA (2–5 μg) was loaded into a 0.8 % agarose gel containing 1.8 % formaldehyde. RNA was transferred to a positively charged nylon membrane (Roche) by capillary blotting and crosslinked by UV irradiation. The probe recognizing all three *C9ORF72* isoforms was synthesized with T7 RNA polymerase (Roche) from cDNAs obtained by PCR with specific primers (Table S1). RNA probes specifically detecting V2 and V3 isoforms were chemically synthesized and 5′ modified to add Dig label (sequence in Table S1). Hybridization was performed overnight at 62 °C.

### Southern blot

Southern blot analysis was performed as described [12] with small modifications. Briefly, genomic DNA (10 μg) was digested overnight with *Xba*I, separated by electrophoresis on a 0.8 % agarose gel, transferred to a positively charged nylon membrane (Roche Applied Science), crosslinked by UV, and hybridized overnight at 47 °C with a digoxigenin-labeled PCR probe. The 676 bp probe was amplified from genomic DNA with specific primers (Table S1) and the PCR DIG Probe Synthesis Kit (Roche). The probe was denatured at 95 °C for 5 min and added to the hybridization mix (EasyHyb granules, Roche). The digoxigenin-labeled probe was detected with anti-digoxigenin antibody and CDP-Star reagent as recommended by the manufacturer (Roche).

### Fluorescence in situ hybridization

FISH was performed using a Cy3-conjugated (GGCCCC)_4_ or (CAGG)_6_ oligonucleotide probes. Briefly, cells on glass coverslips were fixed in 4 % paraformaldehyde for 20 min, permeabilized in 70 % ethanol at 4 °C, incubated with 40 % formamide/2X SSC for 10 min at room temperature, and hybridized for 2 h at 37 °C with a Cy3-conjugated (GGCCCC)_4_ probe (16 ng/ml) in hybridization buffer consisting of 40 % formamide, 2X SSC, 10 % dextran sulfate, yeast tRNA (1 mg/ml), and salmon sperm DNA (1 mg/ml). The cells were washed once with 40 % formamide/1X SSC for 30 min at 37 °C and twice with 1X SSC at room temperature for 30 min. For the RNase A experiments, after fixation, cells were incubated with 24 μg/ml RNase A for 20 min at room temperature.

### Stress-induced toxicity and caspase-3 activity assays

Two-week-old neurons were exposed to: tunicamycin, rotenone, staurosporine, chloroquine, 3-methyladenine or DMSO for 24 h. Cell viability and caspase-3-like activity were determined as described [3]. Cell viability values were expressed as the percentage of the untreated cells or cells treated with DMSO (control) and caspase-like activity was calculated as the increase above control (untreated cells).

### Western blotting

Fifteen micrograms of protein were separated by SDS-PAGE followed by immunoblotting with mouse anti-p62 (1:500, BD Biosciences), mouse anti-Glyceraldehyde-3-Phosphate Dehydrogenase (GAPDH, 1:3,000, Millipore) and HRP-conjugated anti-mouse second antibody (1:5,000). Immunoblots were developed by SuperSignal West Pico Chemiluminescent substrates (Thermo Scientific).

### Pull-down of GGGGCC-binding proteins from mouse brain lysates

300 μg of nuclear extract from mouse brain was passed over an in vitro transcribed and biotinylated (Biotin 11 CTP, Perkin Elmer) (GGGGCC)_30_ RNA bound to streptavidin coated magnetic beads (Dynabeads M-280 streptavidin, Invitrogen) in the presence of 20 mM Hepes, 300 mM NaCl, 2 mM MgCl2, 0.01 % NP40, 1 mM DTT and protease inhibitor (PIC, Roche). The magnetic beads with immobilized RNA and its bound proteins were washed three times with the binding buffer and bound proteins were eluted by boiling 3 min the in sample buffer prior to 4–12 % SDS-PAGE (NuPAGE 4–12 % Bis–Tris Gel, Invitrogen) separation and silver staining (SilverQuest, Invitrogen). The protein bands were excised digested and identified using NanoESI_Ion Trap (LTQ XL Thermo Fisher Scientific).

### Detection of RAN translation products

Sequential extractions of neuronal cell pellets were performed as described by Winton et al. [48]. Human neuronal cell pellets were lysed in cold RIPA buffer and sonicated on ice. Cell lysates were then cleared by centrifugation at 100,000 g for 30 min at 4 °C. The supernatant was collected and protein concentration was determined by BCA assay. To prevent carry-over, the resulting pellet was resuspended in RIPA buffer, re-sonicated and re-centrifuged. The RIPA-insoluble pellet was then extracted using 7 M urea buffer (volume of buffer adjusted based on protein concentration of RIPA soluble samples), sonicated and centrifuged at 100,000 g for 30 min at 22 °C. The protein concentration of the urea soluble supernatant was determined by Bradford assay. Two micrograms of urea soluble sample was directly dotted onto a nitrocellulose membrane, which was then dried, blocked, and finally probed with anti-GP rabbit polyclonal antibody. The anti-GP antibody was generated by immunizing a rabbit with C-Ahx-GPGPGPGPGPGPGPGP-amide. Specificity of antibody was verified by immunoassay and by Western blot using previously described methods [7]. First, (GP)8 peptide or (GR)8 peptide (negative control) were diluted in Tris-buffered saline (TBS) and added to duplicate wells (30 μl/well) of a 96-well Meso Scale Discovery (MSD) assay plate. Following overnight incubation at 4 °C, wells were washed with TBS containing 0.2 % Tween 20 (TBSTw) and blocked with TBSTw + 3 % non-fat milk. Blocking buffer containing anti-GP and SULFO-TAG™-rabbit secondary antibody was then added at 25 μl/well. Following a 2 h incubation and final washes, anti-GP binding to immobilized peptides was evaluated by adding MSD Read Buffer and measuring light emission at 620 nm upon electrochemical stimulation using the MSD Sector Imager 2,400.

For Western blotting, HEK293T cells were transfected using LipofectamineTM 2000 with pEGFP plasmids into which oligonucleotides of 5 repeats of GP, GR, or GA were inserted. Cell lysates were resolved by 10 % Tris–Glycine SDS-PAGE (Invitrogen) and transferred to nitrocellulose membranes for probing with anti-GP. Blots were stripped and reprobed for GFP (Zymed). Pre-immune serum was tested against the peptide antigen and confirmed negative.

### Statistical analysis

Values are expressed as mean ± SEM. The significance of differences among multiple groups was determined with a one-way analysis of variance (ANOVA) followed by a Tukey–Kramer post hoc test (GraphPad Prism version 6.02). Differences were considered significant at *p* < 0.05.

## Results

### Generation and characterization of iPSCs from two members of the VSM-20 family

Two members of the VSM-20 family with FTD/ALS linked to chromosome 9p [8, 12] consented to skin biopsy. To reprogram primary fibroblasts into putative pluripotent stem cells, we transduced the cells with four transcription factors (OCT3/4, SOX2, KLF4, and CMYC) as described [3, 44]. Five weeks after transduction, 20 embryonic stem cell-like colonies per individual were picked and propagated under feeder-free conditions.

**Fig. 1:**
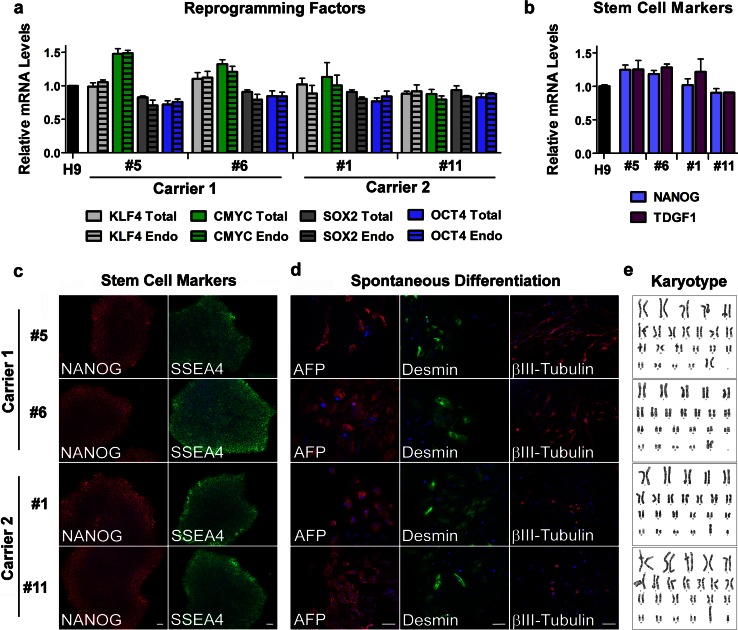
Generation and characterization of iPSC lines from carriers of *C9ORF72* expanded repeats. Total and endogenous (Endo) mRNA levels of the reprogramming factors KLF4, CMYC, SOX2, and OCT4 in iPSC lines from carriers 1 and 2 relative to the values in human embryonic stem cell line H9 were assessed by qRT-PCR. Values are mean ± SEM (**a**). The expression of the pluripotency markers NANOG and TDGF1 (or CRIPTO) was measured at the mRNA level. The values from H9 cells were set to 1. Values are mean ± SEM (**b**). Immunofluorescence analysis of pluripotency markers NANOG and SSEA4 in iPSC lines from carriers 1 and 2 is shown and cell nuclei were counterstained with Hoechst (*blue*). *Scale bar* 50 μm (**c**). After in vitro spontaneous differentiation of iPSC lines into cells of three embryonic germ layers, cells were immunostained with α-fetoprotein (AFP, endoderm), desmin (mesoderm), βIII-tubulin (ectoderm), and Hoechst (nuclei). *Scale bar* 50 μm (**d**). All iPSC lines maintained a normal karyotype (**e**)

Silencing of the transduced transcription factors after reprogramming was confirmed by quantitative RT-PCR (qRT-PCR). Silencing was complete when the total expression level of each factor was not different from that of the endogenous gene. Based on this analysis, two lines from each carrier were selected for further characterization: lines 5 and 6 from VSM-20 family member 1, and lines 1 and 11 from member 2 (Fig. 1a). All four lines also expressed the embryonic stem cell markers *OCT3/4*, *SOX2*, *NANOG*, and teratocarcinoma-derived growth factor 1 (*TDGF1*, or *CRIPTO*) at levels similar to those in human embryonic stem cell line H9 (Fig. 1a, b). Immunofluorescence staining showed high-level expression of the pluripotency markers SSEA4 and NANOG (Fig. 1c). The pluripotency of the iPSCs was also evaluated in vitro through the formation of EBs. All four iPSC lines spontaneously differentiated into cell types of the three embryonic germ layers, as indicated by expression of the specific markers α-fetoprotein (AFP, endoderm), desmin (mesoderm), and βIII-tubulin (ectoderm) (Fig. 1d). Moreover, all these iPSC lines had a normal karyotype (Fig. 1e). These findings indicate successful reprogramming of fibroblasts with *C9ORF72* repeat expansion to a pluripotent state. All the experiments below used iPSC lines between passage 24 and 34.

### Evidence for GGGGCC repeats instability during iPSC reprogramming

**Fig. 2:**
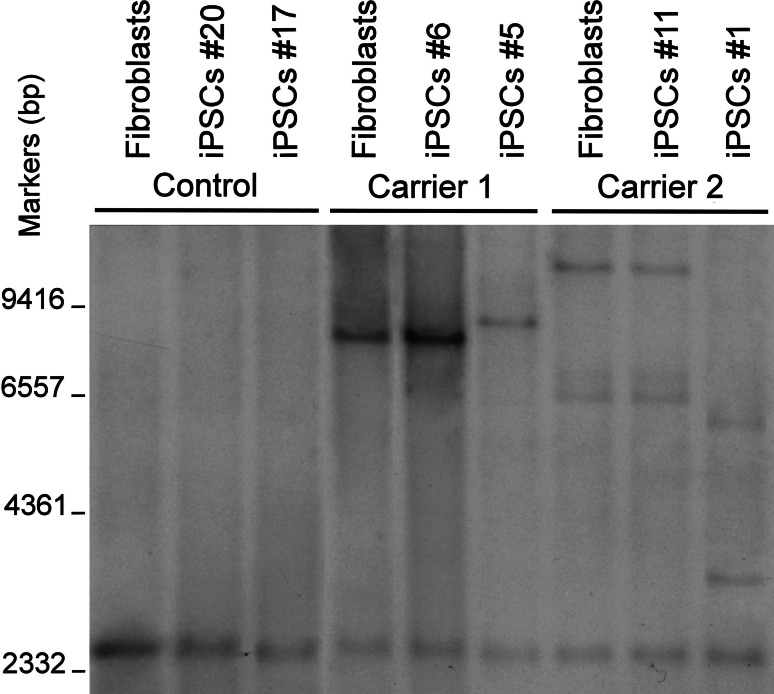
Southern blot analysis of *C9ORF72* alleles in fibroblasts and two iPSC lines (passage 24) derived from each individual. One representative blot is shown here

After the publication of studies showing that GGGGCC repeat expansion is the disease-causing mutation in the VSM-20 family and other patients with FTD/ALS [12, 39], we first performed southern blot analysis to confirm the presence of the repeat expansion in skin fibroblasts from these two members of the VSM-20 family. Consistent with results in lymphoblasts of other VSM-20 family carriers [12], fibroblasts from both family members had both wildtype (2.3 kb) and expanded alleles (Fig. 2). While fibroblasts from 5 healthy and FTD patients without repeat expansion had wildtype allele only on the southern blot (data not shown). Thus, we designated them *C9ORF72* mutation carriers. Carrier 1 had a single mutant band corresponding to ~1,000 repeats. Carrier 2 had three mutant bands, estimated to contain ~1,600, 730, and 650 repeats, suggesting mixed populations of fibroblasts harboring repeats of different lengths. In this experiment and all of the following experiments, iPSCs derived from a healthy control and a sporadic FTD subjects were used which were already published recently [3]. CAG repeat expansion in ataxin 2 is associated with ALS in some patients [17]. We found the number of CAG repeats in both carriers was similar to that in controls (data not shown), ruling out the possibility of a general repeat expansion in different genes in these carriers. After reprogramming of fibroblasts, the expanded GGGGCC alleles were also found in all the iPSCs lines we analyzed but not in control iPSCs (Fig. 2).

To determine the repeat length in iPSCs, we first compared fibroblasts with the corresponding derived iPSC lines for each carrier. For carrier 1, reprogramming did not seem to affect repeat length in line 6, as the band corresponding to the expanded allele has the same size as the band for fibroblasts. The presence of a single predominant repeat size in carrier 1 fibroblasts and iPSC line 6 suggests a certain degree of intrinsic stability. However, in line 5, the repeat length was about 200 repeats longer than the length in the fibroblasts (Fig. 2). Since Southern blot can only detect the most abundant species, and each iPSC line is clonally derived from one single fibroblast, line 6 was most likely originated from one fibroblast containing the more abundant repeat size, whereas line 5 could have been originated from a fibroblast containing a rarer repeat size. Nevertheless, it is also possible that the change in repeat length in line 5 occurred due to instability of the expanded allele during iPSC reprogramming. The latter possibility is supported by the observation of iPSCs derived from carrier 2 (Fig. 2). Line 11 of carrier 2 had the same pattern and repeat lengths as the parental fibroblasts, whereas the repeat length of multiple bands was greatly reduced in line 1. Since a single fibroblast contains only one mutant allele with one defined repeat length, the population of iPSCs with either similar (line 11) or different (line 1) allele combinations from the original population of fibroblasts is most likely generated due to regulated instability of the expanded allele during clonal expansion.

### Differentiation of iPSCs with GGGGCC repeat expansions into functional neurons

**Fig. 3:**
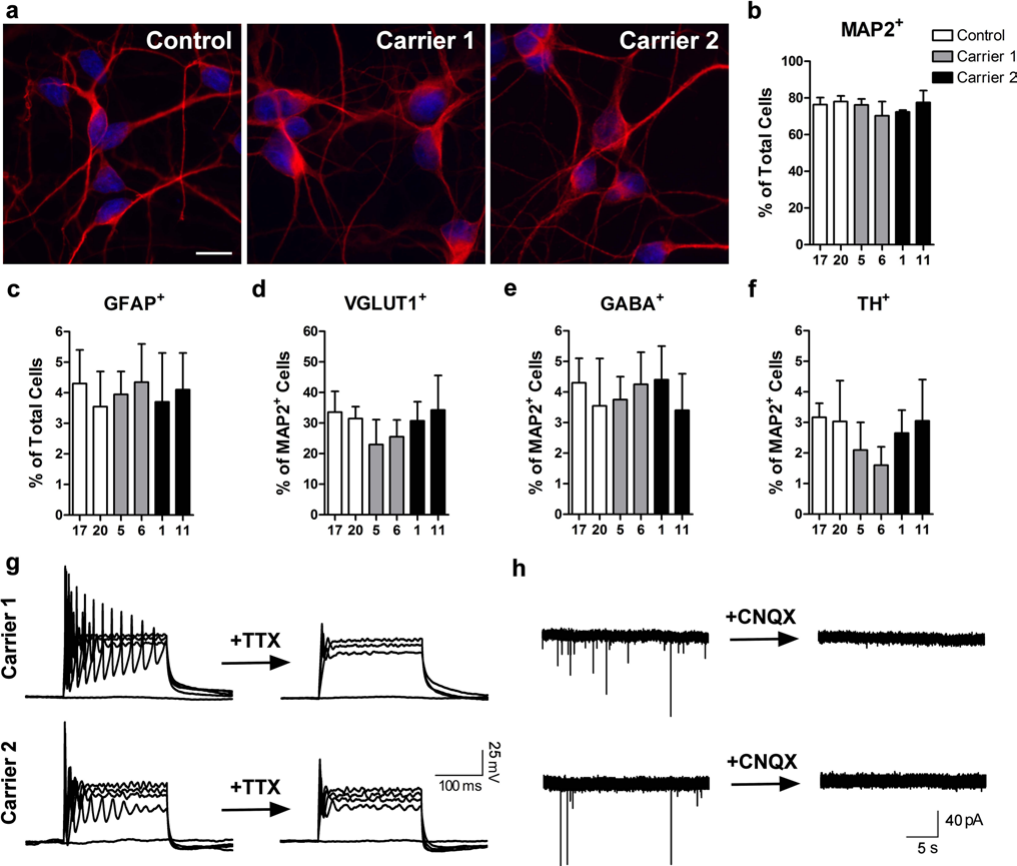
GGGGCC repeats do not affect neuronal differentiation of iPSCs. MAP-2-positive neurons with or without GGGGCC repeat expansions after neuronal differentiation, using methods described in Almeida et al. [3]. Nuclear staining is shown in *blue*. *Scale bar* 50 μm (**a**). The percentage of MAP-2-positive neurons (**b**) and GFAP-positive astrocytes (**c**) in 2 week-old cultures are shown. Cells positive for VGLUT1 (**d**), GABA (**e**), and TH (**f**) (glutamatergic, GABAergic and dopaminergic markers, respectively) were counted as a percentage of MAP-2^+^ cells. On average, 200 cells were analyzed per experiment (*n* = 3 independent cultures). Values are mean ± SEM. Representative depolarization-evoked action potentials from neurons at baseline and after 2 min application of tetrodotoxin (0.5 μM) are shown. Action potentials were elicited by current injections from +400 to 0 pA in 100 pA steps (*n* = 10–14 for each line) (**g**). Representative spontaneous EPSCs at baseline and after 2 min application of CNQX (10 μM). Neurons were held at −60 mV under voltage-clamp (*n* = 10–11 from each line) (**h**)

The neuronal differentiation protocol for iPSC lines was identical to that in our study on human embryonic stem cells [13] and progranulin iPSCs [3]. In order to model FTD, we used this protocol to generate postmitotic neurons derived from neuronal progenitor cells that express the telencephalon marker BF1 (FOXG1) [29]. We did not observe any obvious difference in the neuronal differentiation potential between iPSCs with repeat expansions and controls (Fig. 3a). The percentage of cells positive for the neuronal marker microtubule-associated protein 2 (MAP2, approximately 80 %) or the glial marker glial fibrillary acidic protein (GFAP, <5 %) was similar in controls and repeat carriers (Fig. 3b, c). In addition, we determined whether the presence of GGCCCC repeats affects the type of neurons obtained with this protocol. More than 30 % of the MAP2^+^ cells were also positive for the glutamatergic marker, VGLUT1 (Fig. 3d), and <10 % of cells were GABA^+^ inhibitory neurons or TH^+^ dopaminergic neurons (Fig. 3e, f). Because there was no significant difference between the percentages of neurons differentiated from control and repeat carriers, we concluded that the repeat expansions do not significantly interfere with this neuronal differentiation process.

Similar to what we have previously shown for control neurons [3], neurons from GGGGCC repeat carriers displayed depolarization-evoked action potentials that could be blocked by tetrodotoxin (TTX) (Fig. 3g). In addition, these cells were capable of establishing functional synaptic connections as indicated by the inhibition of spontaneous AMPA-type glutamate receptor-mediated excitatory postsynaptic currents (EPSCs) by CNQX (Fig. 3h) and the presence of PSD95 puncta on dendrites of these neurons (Fig. S1a). The repeat expansion does not seem to affect PSD95 mRNA level (Fig. S1b) or the number of PSD95 puncta (Fig. S1c). The effects of both TTX and CNQX could be reversed after washout (data not shown). These results indicate that postmitotic neurons differentiated from iPSCs with *C9ORF72* repeat expansions are functional.

**Fig. 4:**
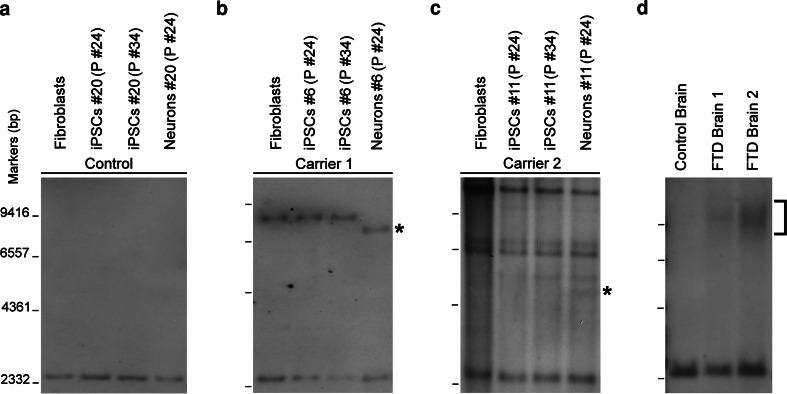
GGGGCC repeats instability during neuronal differentiation of iPSCs. Southern blot analysis of iPSC line 20 of control (**a**), line 6 of carrier 1 (**b**), and line 11 of carrier 2 (**c**) at different passages and their differentiated neurons. The *asterisks* indicate the mutant *C9ORF72* alleles in neurons. Brains from a control subject and two *C9ORF72* repeat expansion carriers show repeats of different lengths (**d**)

We next examined the stability of the GGGGCC repeats during neuronal differentiation of iPSCs. Although little or no change in repeat length was observed during multiple passages of established iPSCs (from 24 to 34 passages), remarkably, the repeat number decreased to ~900 in neurons differentiated from iPSC line 6 of carrier 1 (Fig. 4b). Moreover, one new band corresponding to 400 repeats appeared in neurons differentiated from iPSC line 11 of carrier 2 (Fig. 4c). In both cases, iPSCs at passage 24 were used. Although it remains to be examined to what extent other iPSC lines exhibit such a repeat instability during neuronal differentiation, the repeat instability during neuronal differentiation may partially explain the mosaicism in repeat length in the brains of two FTD patients harboring GGGGCC repeat expansion (Fig. 4d).

### RNA foci and RAN translation products are present in iPSC-derived human neurons

**Fig. 5:**
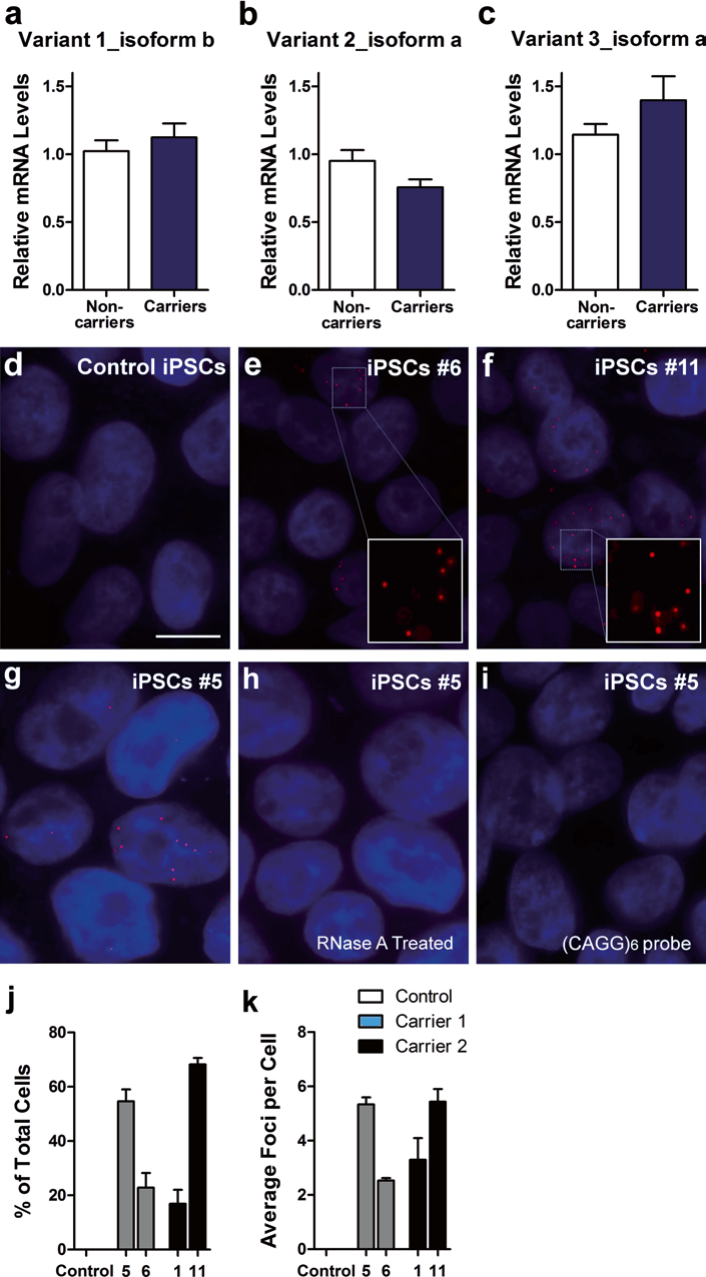
C9ORF72 repeat expansions form RNA foci in iPSCs. Expression levels of *C9ORF72* variant 1 (NM_145005.5, isoform b) (**a**), variant 2 (NM_018325.3, isoform a) (**b**) and variant 3 (NM_001256054.1, isoform a) (**c**) in iPSC lines from two non-carriers and two expanded repeat carriers were assessed by qRT-PCR. Fluorescence in situ hybridization (FISH) analysis was done on control iPSC line 20 (**d**), carrier 1 line 6 iPSCs (**e**), carrier 2 line 11 iPSCs (**f**) using a cy3-conjugated (GGCCCC)_4_ probe. RNA foci (*red*) were found in the nucleus (*blue*) of carriers 1 and 2 but not in control cells. Treatment of iPSCs with RNase A after fixation leads to loss of foci (**h**), indicating that the foci are indeed made of RNA. Representative images of iPSCs from carrier 1 (line 5) that were left untreated (**g**) or treated with RNase A (**h**) for 20 min at room temperature. *Red* RNA foci containing GGGGCC repeats; *blue* nuclei (DAPI). Cells did not show foci when a Cy3-conjugated (CAGG)_6_ probe was used as the negative control probe (**i**). *Scale bar* 10 μm. Quantifications of the percentage of iPSCs displaying foci (**j**) and the average number of foci per cell (**k**) are shown as mean ± SEM

**Fig. 6:**
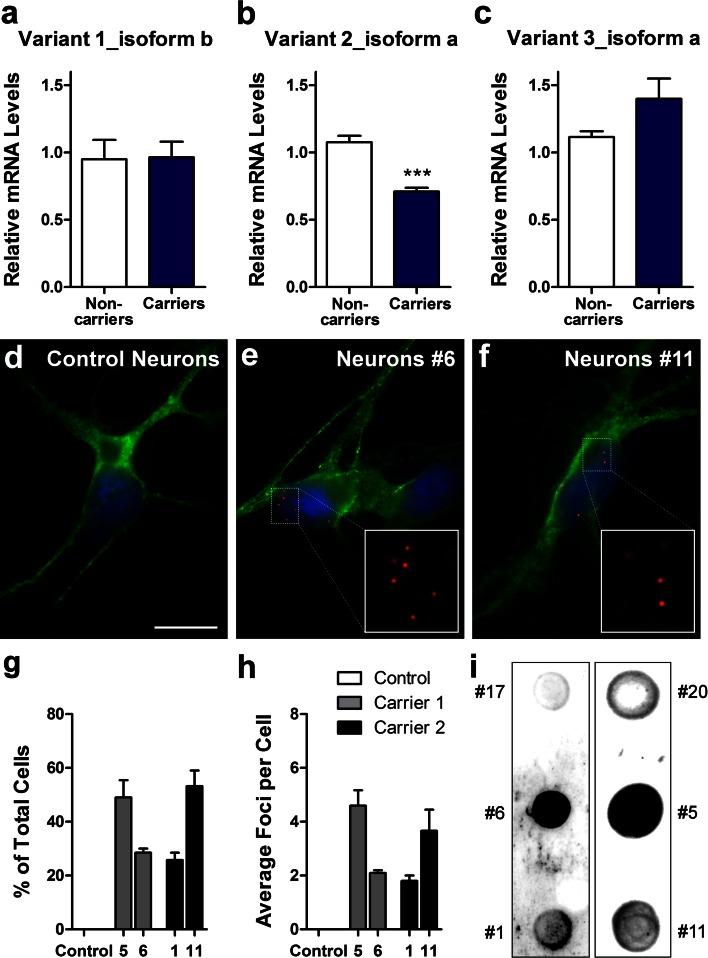
*C9ORF72* repeat expansions form RNA foci in patient iPSCs-derived neurons. Expression levels of *C9ORF72* variant 1 (NM_145005.5, isoform b) (**a**), variant 2 (NM_018325.3, isoform a) (**b**) and variant 3 (NM_001256054.1, isoform a) (**c**) in iPSC-derived neurons from two non-carriers and two expanded repeat carriers were assessed by qRT-PCR. Values are mean ± SEM, *** *p* < 0.001 (Student’s *t* test). FISH analysis was done on control iPSC-derived neurons (**d**), carrier 1 line 6 iPSC-derived neurons (**e**), carrier 2 line 11 iPSC-derived neurons (**f**) using a cy3-conjugated (GGCCCC)_4_ probe. *Green* MAP2. *Blue* DAPI. *Scale bar* 10 μm. Quantifications of the percentage of neurons displaying foci (**g**) and the average number of foci per cell (**h**) are presented as mean ± SEM, based on analysis of neurons derived from three independent differentiation experiments. Gly-Pro dipeptide repeats are detected by dot blot analysis in neurons of carrier 1 (iPSC lines 5 and 6) and carrier 2 (iPSC lines 1 and 11) (**i**)

Nuclear RNA foci containing GGGGCC repeats were found in a subset of brain and spinal cord neurons of FTD/ALS patients with *C9ORF72* repeat expansion but others failed to do so [12, 41]. To resolve this controversial issue, we examined iPSCs and human neurons containing GGGGCC repeat expansions by fluorescence in situ hybridization (FISH). First, we confirmed the expression of different *C9ORF72* RNA variants in iPSCs and iPSC-derived human neurons. The nomenclature of these variants follows the latest in the PubMed in which variant 1 (V1, NM 145005.5) corresponds to the mRNA encoding a truncated C9ORF72 protein (isoform b), and variants 2 and 3 (V2, NM 018325.3 and V3, NM 001256054.1) each encode the full length C9ORF72 protein (isoform a). The hexanucleotide repeats are located in the 5′ end of V2 and in the first intron of V3. We found that V2 and V3 are expressed in iPSCs (Fig. 5a–c, Fig. S2) and iPSC-derived human neurons (Fig. 6a–c). Consistent with a previous report [12], V2 expression was slightly lower in neurons of repeat expansion carriers than that in two non-carriers (Fig. 6b). We also found that on northern blot, neither V1 nor any abnormal mRNA species with larger sizes was detectable in iPSCs (Fig. S2).

Since *C9ORF72* RNA transcripts are expressed in iPSCs and iPSC-derived human neurons, we next determined whether RNA foci containing GGGGCC repeat expansions could be detected. Using a Cy3-conjugated (GGCCCC)_4_ oligonucleotide probe, we detected repeat-containing RNA foci in all iPSC lines from carrier 1 (Fig. 5e, showing representative image from line 6) and carrier 2 (Fig. 5f, representative image from line 11); but no foci were detected in control iPSCs (Fig. 5d). Similarly, these foci were detected in neurons derived from iPSC line 6 from carrier 1 (Fig. 6e) and line 11 from carrier 2 (Fig. 6f) but not in control neurons (Fig. 6d). We confirmed that foci are indeed made of RNA as after RNase treatment no foci could be detected (Fig. 5g, h). To confirm that these foci are specific to the (GGCCCC)_4_ oligonucleotide probe, we used a (CAG)_8_ probe specific for CTG repeats associated with myotonic dystrophy type 1 (data not shown) and a (CAGG)_6_ probe specific for CCTG repeats associated with myotonic dystrophy type 2. No foci were detected in iPSCs (Fig. 5i) or iPSC-derived human neurons (data not shown). To further demonstrate that these RNA foci are specific to carriers with GGGGCC repeat expansions, we examined primary fibroblasts from 5 carriers and 5 subjects with no disease or other FTD mutations. RNA foci containing GGGGCC repeats were found in some fibroblasts of all five carriers but were absent in controls or patients with FTD due to mutations in *MAPT* or *GRN* (Fig. S3).

Discrete and punctate RNA foci containing GGGGCC repeats were mostly found in the nucleus. Occasionally a few foci were seen outside the nucleus, as in other repeat diseases [47]. These RNA foci are similar in size to PML nuclear bodies but they do not overlap (Fig. S4). Because GGGGCC-containing RNA foci are not present in all iPSCs or human neurons and are relatively small in size, it is unlikely these foci in human cells under these culture conditions can impair RNA metabolism through sequestering sufficient amounts of some abundantly expressed nuclear RNA-binding proteins. Indeed, among the top 30 RNA-binding proteins that we identified in mouse brain extracts to bind to biotinylated (GGGGCC)_30_ RNA in vitro, 8 are hnRNP proteins. We found H1 (Fig. S4) and H2 (Fig. S5) were not sequestered into the foci or mislocalized in iPSCs, even though transiently overexpressed GGGGCC repeats could artificially sequester these proteins under non-physiological conditions (Fig. S6). Other major RNA/DNA-binding proteins of interest include FUS, TDP-43, NONO, FMRP, nucleolin and nucleophosmin. The latter two interact with each other and are known to bind to G-rich RNAs and DNA G-quadruplexes [1, 10]. Interestingly, recent studies suggest that GGGGCC repeats associated with FTD/ALS form RNA G-quadruplexes [18, 38]. However, nucleophosmin was not sequestered in GGGGCC repeats-containing foci with seemingly normal subcellular distribution in iPSCs (Figs. S4, S5) or in human neurons or primary fibroblasts derived from GGGGCC repeat expansion carriers (data not shown). Similarly, no change in subcellular localization was observed for FUS, TDP-43, hnRNPA2/B1, hnRNP F, and other RNA-binding proteins in iPSCs (Figs. S4, S5). Thus, these foci may not sequester sufficient quantity of these or other abundant nuclear RNA/DNA-binding proteins to cause their mislocalization in these patient cells, at least under the culture conditions used here.

Repeat-containing RNA foci were not found in all cells. For instance, 23 % of iPSCs from line 6 of carrier 1 and 68 % of those from line 11 of carrier 2 contained foci (Fig. 5j), which were abundant in some iPSCs and scarce in others. Similarly, GGGGCC repeat-containing RNA foci were present in 29 % of MAP2-positive neurons derived from line 6 and 53 % of those from line 11 (Fig. 6g). Although the length of these repeats cannot be manipulated experimentally, we were fortunate to obtain iPSC lines with different repeat lengths (Fig. 2). The repeat length was substantially higher in line 6 than line 1 (Fig. 2), but both the average percentage of iPSCs or neurons with foci (Figs. 5j, 6g) and the average number of foci per cell (Figs. 5k, 6h) were similar. Moreover, the repeat length in line 5 is only slightly higher than that in line 6 (Fig. 2), yet, the percentage of cells with foci (Figs. 5j, 6g) and the average number of foci per cell (Figs. 5k, 6i) are much higher in line 5 than line 6. Since these cells were cultured under identical conditions, our data indicate that the formation of RNA foci containing GGGGCC repeats is determined not simply by the number of repeats but also by other genetic or epigenetic factors in the parental fibroblast and subsequent iPSC clone.

Very recent studies demonstrate that di-peptide repeats can be produced specifically in neurons of patients with expanded GGGGCC repeats [7, 32]. To examine whether this major pathological feature of C9FTD/ALS can also be replicated in iPSC-derived human neurons, we performed dot blot analysis of RIPA-insoluble protein lysates obtained from human neurons with GGGGCC repeat expansions. A new anti-Gly-Pro (GP) antibody was generated and its specificity was demonstrated (Fig. S7). Indeed, we detected very strong expression of GP dipetides in neurons differentiated from two iPSC lines of carrier 1 and to a lesser extent in neurons of carrier 2 (Fig. 6i). There is no direct positive correlation between repeat length, the number of RNA foci and the level of RAN translation; thus, these two major neuropathologic phenotypes seem to be independent of repeat length and of each other.

### Human neurons with GGGGCC repeat expansions are more sensitive to cellular stress induced by autophagy inhibitors

**Fig. 7:**
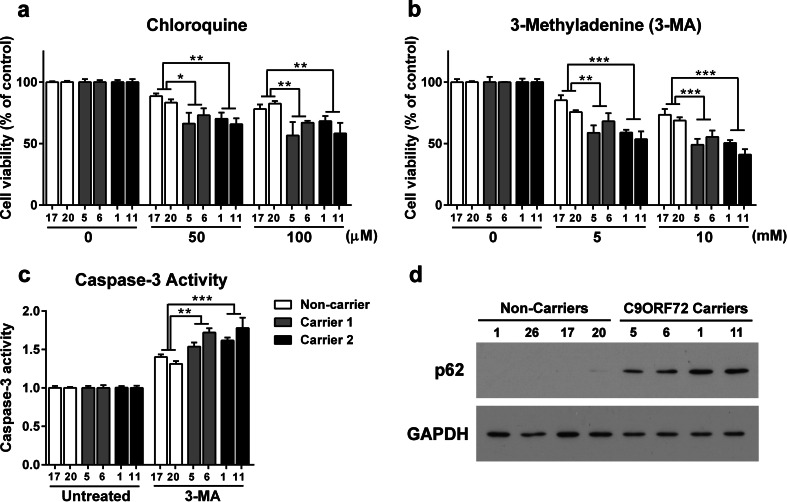
GGGGCC repeat expansions exacerbate susceptibility to inhibition of the autophagy pathway. Cell viability of human neurons after exposure to chloroquine (**a**) and 3-methyladenine (3-MA) (**b**) for 24 h. Values are expressed as a percentage of the untreated cells (control) (*n* = 3 independent cultures) (**a, b**). Caspase-3-like activity after exposure to 5 mM 3-MA for 24 h (**c**). In all panels, values are mean ± SEM. *p < 0.05, **p < 0.01, ***p < 0.001 (One-way ANOVA). Representative Western blot showing p62 protein levels in neurons of two non-carriers (PGRN S116X: lines 1 and 26, and control: lines 17 and 20) and two repeat expansions carriers (carrier 1: lines 5 and 6, and carrier 2: lines 1 and 11) (**d**)

In order to reveal novel pathogenic mechanisms in FTD/ALS with GGGGCC repeat expansions, we performed a systematic screen of several inducers of cellular stress similar to our study on iPSC-derived human neurons with progranulin deficiency [3]. We found that human neurons with GGGGCC repeat expansions were not more sensitive to cellular stress induced by rotenone, a complex I inhibitor and inducer of mitochondrial dysfunction, tunicamycin, an inhibitor of protein N-glycosylation and stressor of endoplasmic reticulum (ER), and staurosporine, a broad-spectrum kinase inhibitor (Fig. S8). Interestingly, chloroquine, an inhibitor of autophagy [5], decreased cell viability of these neurons (Fig. 7a). To confirm this finding, we used another known autophagy inhibitor, 3-methyladenine (3-MA) [28, 40], and found that human neurons with GGGGCC repeat expansions were more sensitive to 3-MA treatment than control neurons (Fig. 7b). To validate the findings of the cell viability assay (Fig. 7b), we also measured the activation of caspase-3 as we did before [3]. Consistent with the results of the cell viability assay, human neurons with GGGGCC repeat expansions showed greater caspase-3 activation in response to 3-MA than control neurons (Fig. 7c). These findings raise the possibility that the autophagy function is compromised in these neurons. To provide further support for this notion, we found that p62, a known substrate of the autophagy pathway that is a component in neuronal inclusions in C9ORF72 patients [4], significantly accumulated in human neurons with GGGGCC repeat expansions but not in neurons derived from control iPSCs or neurons derived from iPSCs of an FTD patient with a progranulin mutation (Fig. 7d).

## Discussion

In this study, we established multiple iPSC lines with over 1000 GGGGCC repeats from two members of the VSM-20 family. The repeat lengths varied among different iPSC lines and changed during neuronal differentiation of a given iPSC line. Thus, we have established a model system for studying repeat instability in the native genetic context. RNA foci containing GGGGCC repeats were present in some iPSCs, iPSC-derived human neurons, and primary fibroblasts of repeat expansion carriers but were not found in cells from healthy subjects or FTD patients with other genetic mutations. Nuclear RNA foci containing GGGGCC repeats do not seem to sequester several major RNA-binding proteins under the culture condition used in this study. Moreover, RAN translation products were detected in iPSCs-derived human neurons with repeat expansion. The abundance of RAN translation products and the percentage of cells with foci were not determined simply by repeat length but probably also by other factors as well. More importantly, our findings on inducers of cellular stress imply that a compromised autophagy pathway may be a novel pathogenic mechanism.

During iPSC reprogramming, fibroblasts were dissociated into single cell suspension and plated at a very low density, so it is thought each iPSC clone is derived from a single fibroblast cell [44]. Thus, it seems certain that line 6 was derived from a fibroblast with a repeat length identical to that of most fibroblasts from the carrier (Fig. 2). The repeat length in line 5 was higher, perhaps because of repeat instability during reprogramming or because the parental fibroblast had a repeat length longer than that of most fibroblasts of the carrier. In contrast, iPSC lines derived from carrier 2 suggest a dynamic instability of GGGGCC repeat expansions during reprogramming. Fibroblasts from carrier 2 contained repeats of at least three different lengths, suggesting a somatic mosaicism. These three alleles seem to be stable and preferred in the fibroblast cell population since no other significant bands are seen on the southern blot. Thus, the genetic makeups of different fibroblasts are not identical. Indeed, human skin fibroblasts show somatic copy number mosaicism [2]. Interestingly, line 1 had multiple alleles with smaller repeat length, strongly indicating repeat instability during reprogramming. A non-clonal origin is highly unlikely since the probability that two adjacent fibroblasts with rare alleles of smaller repeat length were reprogrammed simultaneously is very low. Such a change in repeat length could well occur before the first passage or during very early passages, during which it is impossible to do southern blot analysis to measure the repeat length due to limited amount of samples. Line 11 showed the same complex pattern as the parental fibroblasts (Fig. 2), raising the possibility that, after clonal expansion of a reprogrammed fibroblast with the largest repeat length, the generation of expanded alleles with shorter length within the same population of iPSCs is re-established as that in fibroblasts. Further studies are warranted to completely rule out other possible explanations. However, GGGGCC repeat instability is consistent with varying degrees of instability observed in iPSC models of other repeat diseases [20, 25, 36].

Another major finding in this study is the presence of RNA foci and RAN translation products in iPSCs-derived human neurons, recapitulating two major neuropathological features of FTD patients with *C9ORF72* repeat expansion. Both phenotypes do not seem to show a positive correlation with the repeat length. GGGGCC-containing RNA foci formed only in cells from five *C9ORF72* repeat expansion carriers but not in cells from controls or patients with mutations in *MAPT* or *GRN*. Moreover, the percentage of cells with foci and the average number of foci per cell did not correlate with the maximum length of repeats, suggesting that the detection of these foci is not simply due to the presence of higher copies of repeats and that unknown genetic factors contribute to the formation of these RNA foci. Since whether foci are present in patient brains is controversial [12, 41], the detection of RNA foci in iPSCs-derived patient neurons highlights the importance of understanding these structures.

The RNA foci might exert a toxic gain of function by sequestering RNA-binding proteins, as described in other repeat diseases such as myotonic dystrophies. In contract to large nuclear RNA foci [12] and p62^+^/hnRNP A3^+^ cytoplasmic aggregates in the brains of patients with *C9ORF72* repeat expansion [31], the nuclear RNA foci observed here in iPSCs and iPSCs-derived human neurons are relatively small in size and several abundant nuclear RNA-binding proteins that can bind to and sequester GGGGCC repeats in vitro are not mislocalized, at least under the culture conditions used in this study. Transiently overexpressed GGGGCC repeats did sequester some hnRNP proteins in cultured cells (Fig. S6), which may be an experimental artifact. Thus, it remains to be determined what proteins are sequestered in the GGGGCC-containing RNA foci in human neurons under normal or stress culture conditions and in patient brains. It is possible that in patient brains, the size of RNA foci may increase during disease progression over decades, leading to the sequestration of sufficient quantity of nuclear RNA-binding proteins to impair RNA metabolism.

Another remaining question is whether RAN translation products are toxic per se. Our findings that human neurons with GGGGCC repeat expansions show p62 accumulation and are more sensitive to autophagy inhibitors raise the possibility that RAN translation products may directly or indirectly impair the autophagy pathway. It is interesting to note that other FTD mutant proteins also disrupt this pathway, such as VCP [23] and CHMP2B [27], in which further activation of the autophagy pathway may be detrimental to neuronal survival [28]. The availability of iPSC-derived human neurons with GGGGCC repeat expansions in patients’ native genetic context will serve as a compelling model to further investigate underlying pathogenic mechanisms and broaden our knowledge of this devastating disease.

## Electronic supplementary material

Below is the link to the electronic supplementary material.

Supplementary material 1 (JPG 457 kb)

Supplementary material 2 (JPG 60 kb)

Supplementary material 3 (JPG 1109 kb)

Supplementary material 4 (JPG 608 kb)

Supplementary material 5 (JPG 797 kb)

Supplementary material 6 (PDF 56 kb)

Supplementary material 7 (JPG 57 kb)

Supplementary material 8 (JPG 680 kb)

Supplementary material 9 (DOCX 16 kb)
