# Supplementary figures and images for: Modeling key pathological features of frontotemporal dementia with C9ORF72 repeat expansion in iPSC-derived human neurons
Source: Acta Neuropathol. 2013 Jul 9;126(3):385–99. doi: 10.1007/s00401-013-1149-y (PMC3753484; doi:10.1007/s00401-013-1149-y)

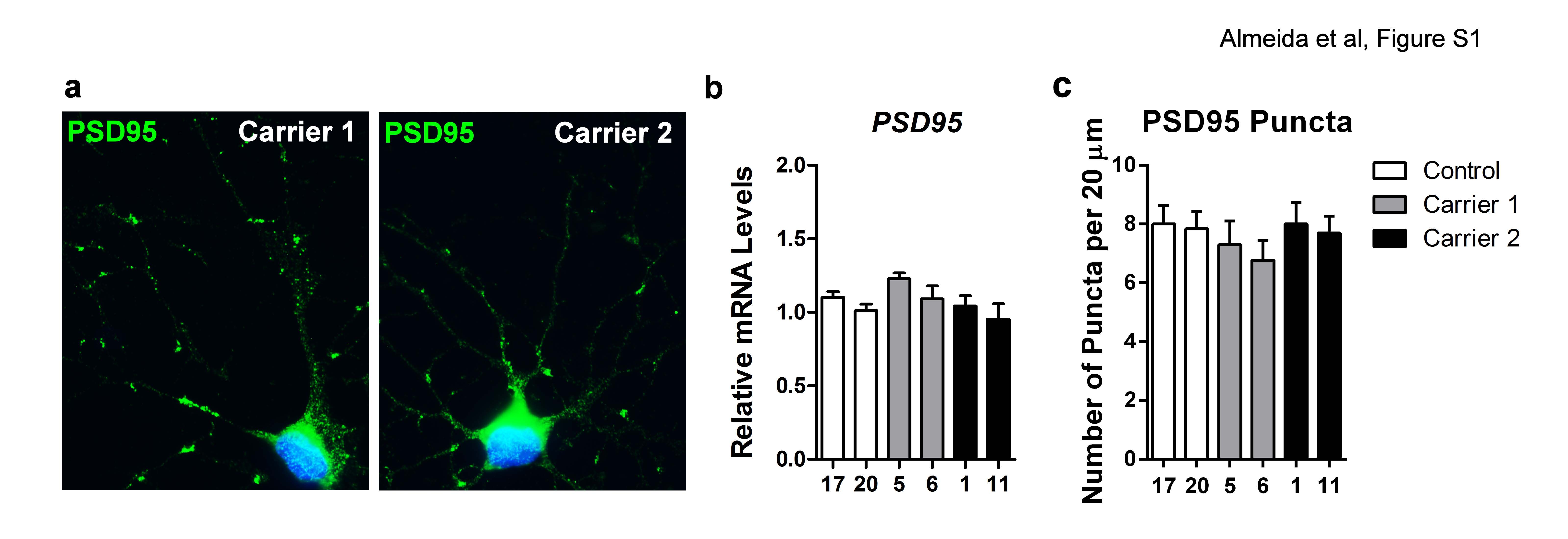

Supplement: Supplementary file 1 — Supplementary material 1 (JPG 457 kb) [file 401_2013_1149_MOESM1_ESM.jpg]

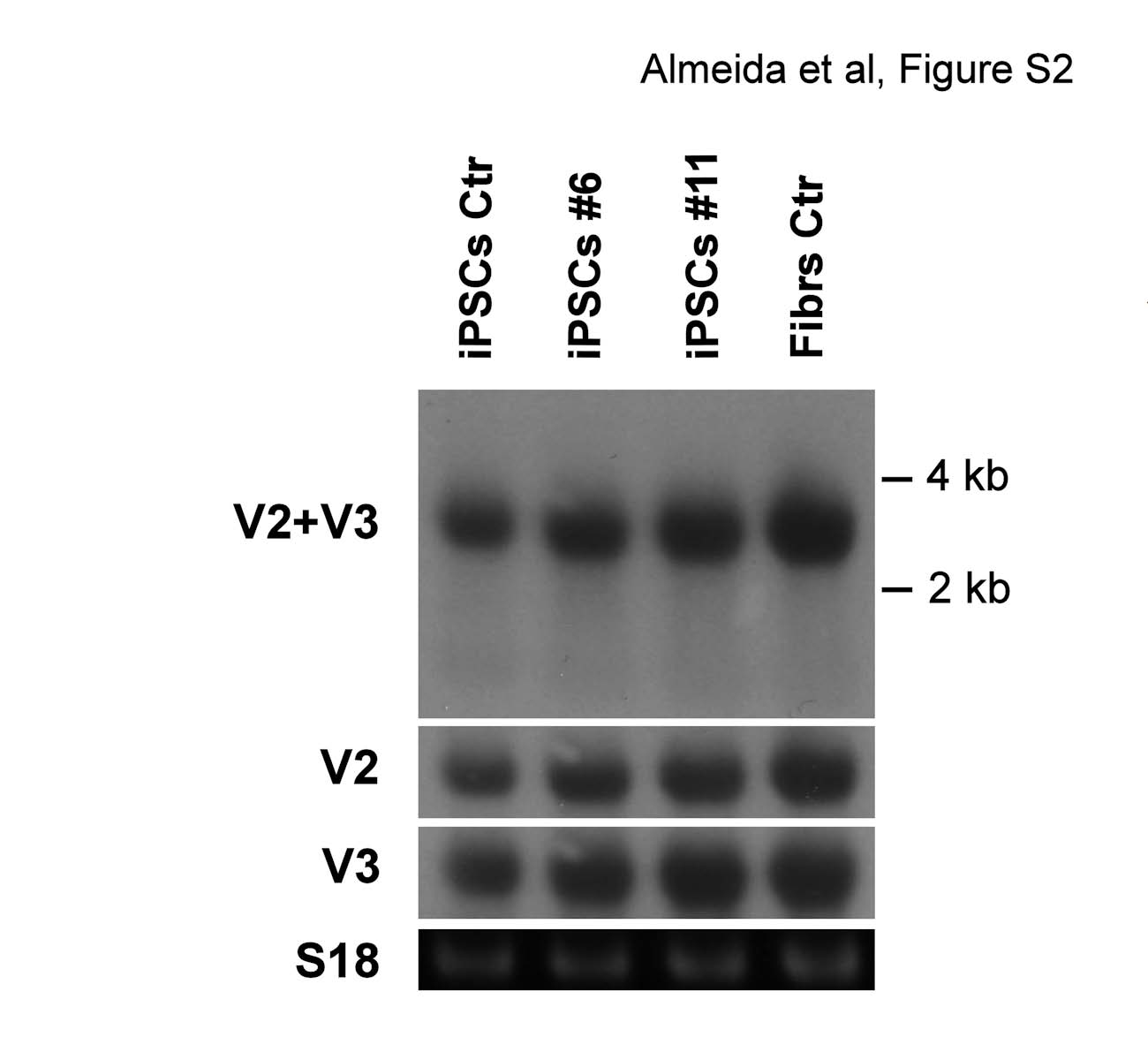

Supplement: Supplementary file 2 — Supplementary material 2 (JPG 60 kb) [file 401_2013_1149_MOESM2_ESM.jpg]

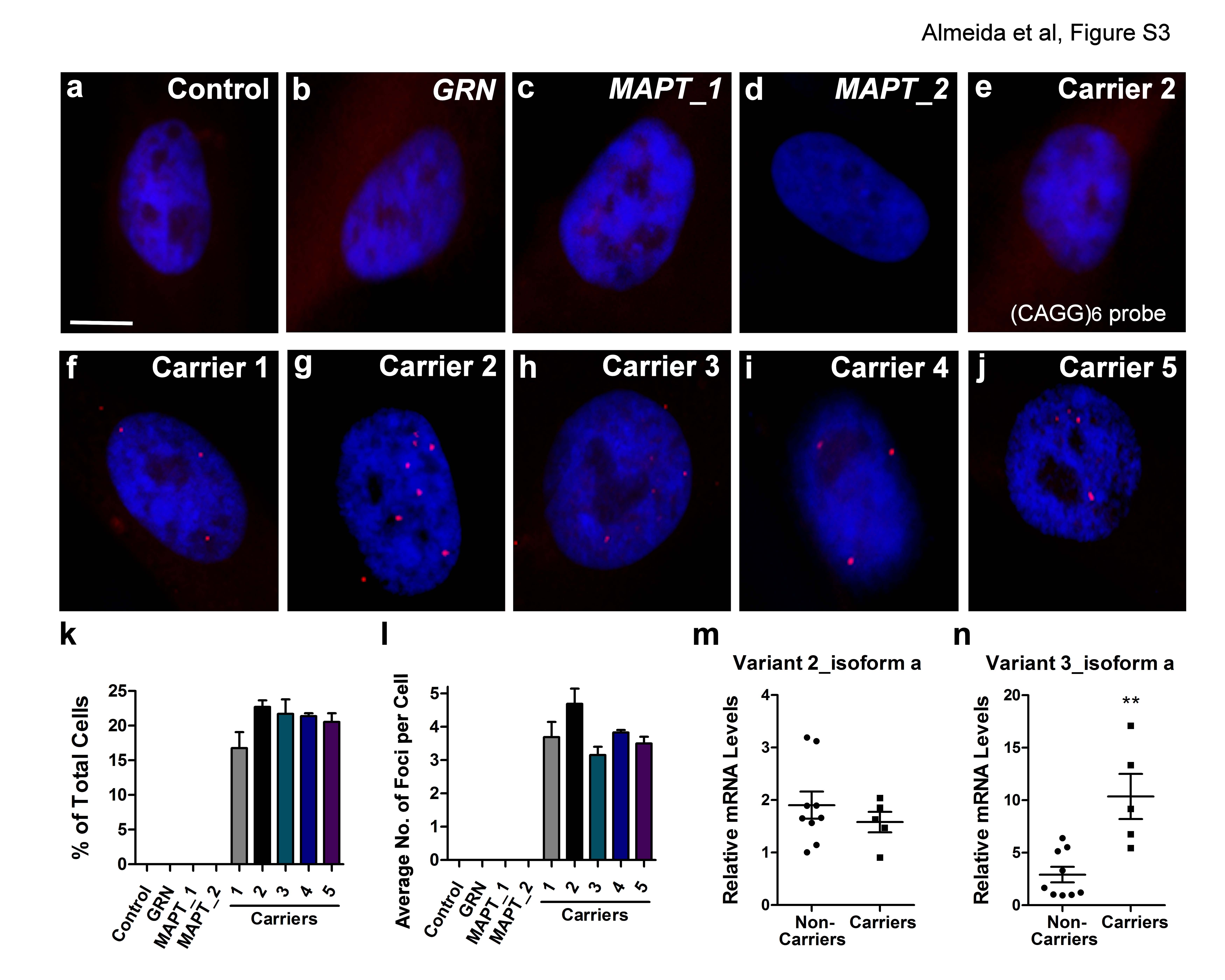

Supplement: Supplementary file 3 — Supplementary material 3 (JPG 1109 kb) [file 401_2013_1149_MOESM3_ESM.jpg]

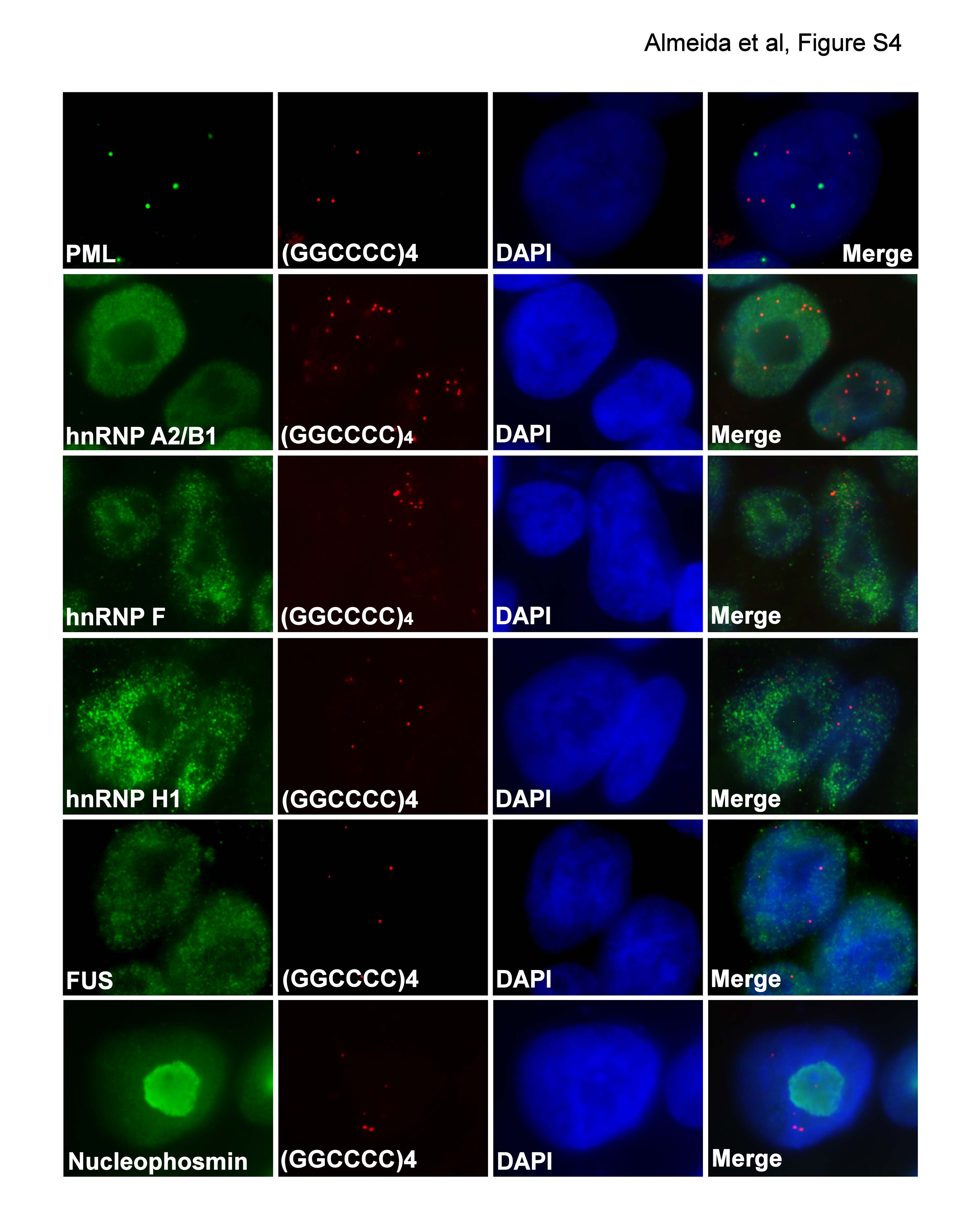

Supplement: Supplementary file 4 — Supplementary material 4 (JPG 608 kb) [file 401_2013_1149_MOESM4_ESM.jpg]

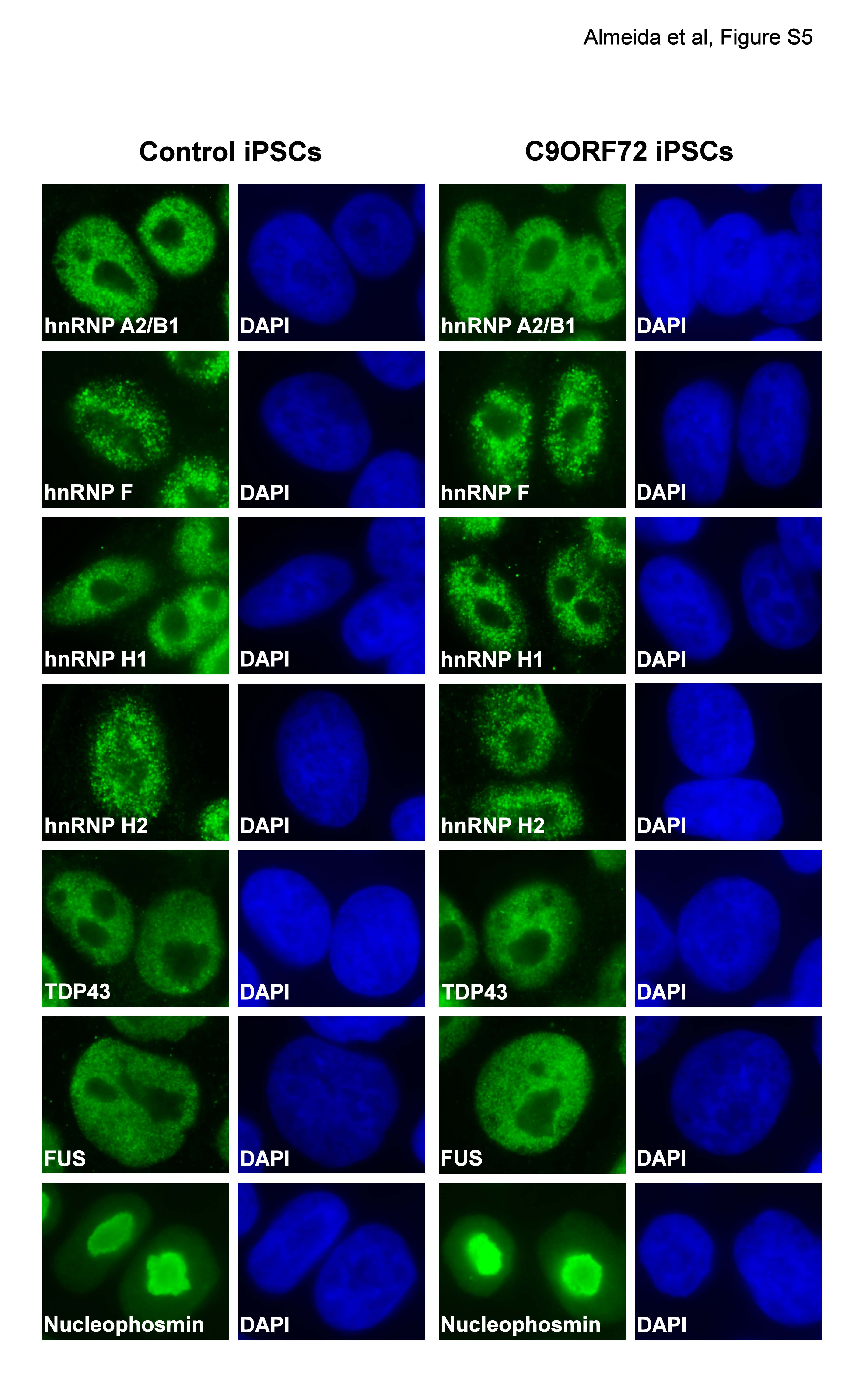

Supplement: Supplementary file 5 — Supplementary material 5 (JPG 797 kb) [file 401_2013_1149_MOESM5_ESM.jpg]

Almeida et al., Figure S6

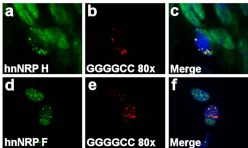

Supplement: Supplementary file 6 — Supplementary material 6 (PDF 56 kb) [file 401_2013_1149_MOESM6_ESM.pdf]

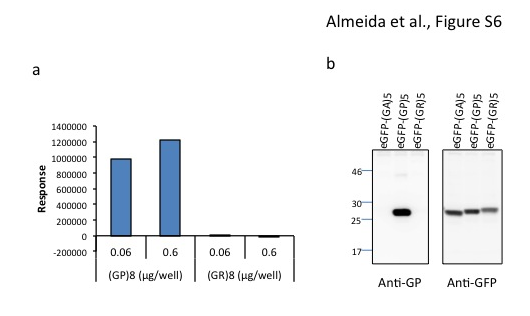

Supplement: Supplementary file 7 — Supplementary material 7 (JPG 57 kb) [file 401_2013_1149_MOESM7_ESM.jpg]

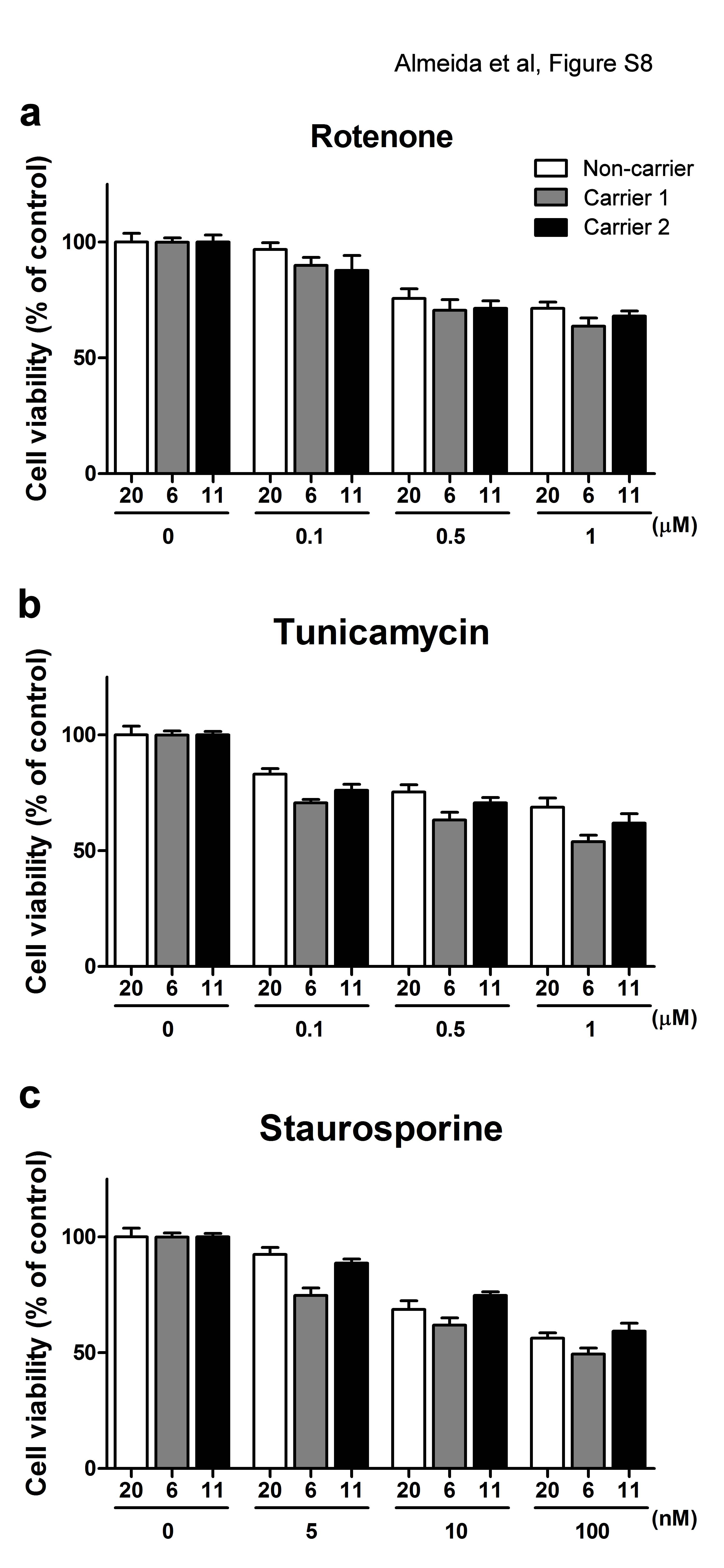

Supplement: Supplementary file 8 — Supplementary material 8 (JPG 680 kb) [file 401_2013_1149_MOESM8_ESM.jpg]
