# Supplementary material for: Modeling key pathological features of frontotemporal dementia with C9ORF72 repeat expansion in iPSC-derived human neurons
Source: Acta Neuropathol. 2013 Jul 9;126(3):385–99. doi: 10.1007/s00401-013-1149-y (PMC3753484; doi:10.1007/s00401-013-1149-y)
Supplement: Supplementary file 9 — Supplementary material 9 (DOCX 16 kb) [file 401_2013_1149_MOESM9_ESM.docx]

**Table S1**. Primer for qPCR and PCR reactions.

| **Gene name** | **Forward primer (5’to 3’)** | **Reverse primer (5’ to 3’)** |
| --- | --- | --- |
| **For qPCR** | | |
| GAPDH | TGCACCACCACCTGCTTAGC | GGCATGGACTGTGGTCATGAG |
| C9ORF72 V2 | CGGTGGCGAGTGGATATCT | GCCCAAATGTGCCTTACTCT |
| C9ORF72 V3 | GGGTCTAGCAAGAGCAGGTG | AGCCCAAATGTGCCTTACTC |
| **For Northern blot** | | |
| C9ORF72 total | TAATACGACTCACTATAGGGTTGGAATGCAGTGATGTCG (T7 promoter underlined) | TTCCATTCTCTCTGTGCCTTC |
| V2 specific | CCACUCGCCACCGCUGCGCCUCCGCCGCCGCGGGCGCAGGCACCGCAAC | |
| V3 specific | ACCCCGCCCCCAAAAGAGAAGCAACCGGGCAGCAGGGACGGCUGACACAC | |
| **For Southern blot** | | |
|  | CTTGCAGATCAAAAGGCACA | TTGACGCACCTCTCTTTCCT |
